# Cardiovascular Pathology in Males and Females with 45,X/46,XY Mosaicism

**DOI:** 10.1371/journal.pone.0054977

**Published:** 2013-02-14

**Authors:** Katya De Groote, Martine Cools, Jean De Schepper, Margarita Craen, Inge François, Daniel Devos, Karlien Carbonez, Benedicte Eyskens, Daniel De Wolf

**Affiliations:** 1 Division of Pediatric Cardiology, Department of Pediatrics, Ghent University Hospital and Ghent University, Ghent, Belgium; 2 Division of Pediatric Endocrinology, Department of Pediatrics, Ghent University Hospital and Ghent University, Ghent, Belgium; 3 Department of Radiology, Ghent University Hospital and Ghent University, Ghent, Belgium; 4 Division of Pediatric Cardiology, Department of Pediatrics, University Hospitals Leuven, Leuven, Belgium; 5 Division of Pediatric Endocrinology, Department of Pediatrics, University Hospitals Leuven, Leuven, Belgium; 6 Division of Pediatric Cardiology, Department of Pediatrics, Cliniques Universitaires Saint-Luc, Brussels, Belgium; Institute for Nanotechnology and Stem Cell Biology, United States of America

## Abstract

**Context:**

The phenotype of 45,X/46,XY mosaicism is heterogeneous ranging from females with Turner syndrome (TS) to apparently normal males. Males with 45,X/46,XY frequently show stigmata typically associated with TS. We hypothesised that males with 45,X/46,XY have similar cardiovascular pathology as females with 45,X/46,XY.

**Objective:**

To investigate cardiovascular abnormalities in 45,X/46,XY males and to compare them with 45,X/46,XY females.

**Design:**

Patients with 45,X/46,XY mosaicism were selected from the Belgian Registry for Growth and Puberty problems and via the multidisciplinary clinic for disorders of sexual development.

**Patients:**

Eighteen patients were included: 8 raised as females (F) and 10 as males (M).

**Intervention:**

Complete cardiac examination with blood pressure measurement, ECG, echocardiography and MRI.

**Main Outcome Measurement:**

Cardiac parameters were registered for both groups. In a second phase, clinical features and external masculinisation score (EMS) were retrospectively collected from the medical files.

**Results:**

A structural heart defect was diagnosed before inclusion in 1 F with coarctation and 1 M with spontaneously closed VSD. A bicuspid aortic valve was found in 8 (3 F, 5 M). Dilation of the ascending aorta was present in 4 M and was severe in 2 young boys. QTc was prolonged in 3 F and 2 M.

**Conclusion:**

Males with 45,X/46,XY mosaicism have similar cardiovascular pathology as 45,X/46,XY females. Dilation of the ascending aorta can be important, also in males. We advise cardiac screening and life-long monitoring in all males with 45,X/46,XY mosaicism according to the existing guidelines for Turner syndrome.

## Introduction

Turner syndrome (TS) is a genetic disorder, occurring in approximately 50 per 100 000 live born girls [1]. Monosomy X is present in about 50% of the cases. The remaining have a mosaic karyotype, consisting of a 45,X cell line in combination with at least one of the following : 46,XX (or variants), 47,XXX (or variants), or a whole or partial Y chromosome [1], [2]. Cardinal features of TS include reduced final height and gonadal failure. Diabetes, hypothyroidism, hearing disorders, scoliosis, renal abnormalities and neurocognitive disorders are frequently associated pathologies [3]. Compared to the normal population, morbidity and mortality are high, circulatory disease accounting for the greatest excess mortality [4]–[6]. Structural heart defects are found in one fourth to half of the Turner patients and involve mainly the left side of the heart. Bicuspid aortic valve, coarctation aortae and dilation of the ascending aorta are the most common [3], [7]–[9]. Hypertension occurs in about half of the patients [10], [11]. Current guidelines advocate systematic and repeated cardiac screening in all Turner patients [12].

In about 6% of TS girls, 45,X/46,XY mosaicism is documented [13]. On the other hand, sex chromosome mosaicism (45,X/46,XY and variants) is sometimes diagnosed in apparently normal males presenting at infertility clinics, in boys consulting for short stature or in neonates born with genital ambiguity [14], [15]. Some male patients show stigmata typically associated with TS, e.g. short stature, renal pathology and coarctation [15]. To our knowledge, no data exist on the frequency and nature of cardiovascular pathology in males with 45,X/46,XY.

The present study was undertaken to investigate cardiovascular abnormalities in 45,X/46,XY males and to compare them with 45,X/46,XY girls with TS. In view of the description of some (fatal) cases of aortic dilation in males with 45,X/46,XY mixed gonadal dysgenesis [16], this information is highly relevant, taking into account that current guidelines on regular cardiac follow-up exclusively implicate girls with TS. If similar cardiovascular pathology is encountered in 45,X/46,XY boys and girls alike, consequent adjustment of these guidelines is mandatory.

## Patients and Methods

### Study Population

We identified patients with 45,X/46,XY mosaicism from the Belgian Registry for Growth and Puberty problems. Four Belgian university centres agreed to let their patients take part in a single-centre cardiovascular study. The study protocol was approved by the ethical committee of Ghent University Hospital and additionally by the ethical committee of each participating centre. From the 34 identified patients, 11 accepted to participate in the study protocol. Seven additional patients were included via the multidisciplinary clinic for disorders of sexual development of the Ghent University Hospital. All patients and/or their parents gave written informed consent.

All investigations were performed in one day at Ghent University Hospital from May 2009 to November 2011. Clinical cardiac examination was performed by one experienced paediatric cardiologist (KDG). Height and weight were measured and used to calculate body surface aera (BSA) according to the Haycock’s formula [17].

Blood pressure was taken at the 4 limbs, with the patient at rest and in a supine position (Datascope, Accutorr Plus, USA). In children and adolescents, blood pressure was considered borderline if ≥p95 and high if ≥p97 for age and height [18]. In adults, hypertension was defined as a systolic pressure ≥130 mmHg and/or a diastolic pressure ≥85 mm Hg.

A standard 12 leads ECG (GE Healthcare, Marquette Mac5000, USA) was recorded and analysed by one single observer (KDG). Conform the international guidelines, QTc was manually calculated in at least 3 consecutive heartbeats, based on Bazett’s formula. QTc was considered prolonged if >450 ms [19].

Echocardiography was performed by a single observer (KDG) with the use of a VIVID 7 ultrasound (GE Vingmed Ultrasound, Horten, Norway) equipped with a 3,5 MHz and 7 MHz probe. Evaluation included a complete study with 2D, M-mode and Doppler. Data were stored and used for offline analysis using EchoPAC version 110.1.0 software (GE Vingmed Ultrasound). Diameters of the aorta were measured from the parasternal long axis in systole from inner tot inner surface at the level of the aortic valve annulus, aortic root (AoRt), sinotubular junction (STJ) and ascending aorta (AoAsc). Measurement of the transverse arch was obtained from the suprasternal notch. Measurement of the interventricular septum, posterior wall and internal diameters were performed on a parasternal short axis and used for calculation of fractional shortening. For each variable, z-scores were calculated [20].

Cardiac MRI was performed on a 1.5T magnet (slope 45 mt/m, slewrate: 200T/m/sec) (Siemens Avanto, Erlangen) and analysed by one single observer (DD). ECG gated TrueFISP cine imaging of cardiac long axis and the left ventricle outflow apparatus were acquired. A 3D flash sequence was used before and during intravenous injection of Gadobutol (Bayer Schering) at 1 ml/10 kg bodyweight, using a bolus tracking technique for optimal timing of the acquisition of arterial phase images. Evaluation of aortic valve was done on the cine images. Assessment of the thoracic aorta consisted of multiplanar reconstruction perpendicular to the aortic lumen, and measurement of the aortic diameters: aortic valve annulus, AoRt, STJ and AoAsc. The diameter of the ascending aorta was divided by BSA to correct for the relative short stature of the patients. An ascending aorta/BSA (indexed AoAsc)>20 mm/m^2^ was considered dilated, ≥25 mm/m^2^ severely dilated [21].

Clinical data were collected from the medical records. The genital phenotype of the patients at first examination, if other than typically female, was described by the external masculinisation score (EMS) which represents a clinical scoring system (based on the position of the gonads, length of the phallus, presence of labioscrotal fusion and position of the urethreal meatus) to quantitatively assess the degree of undervirilisation [22].

## Results

### Patients

Eighteen patients were included in the study, of which 8 (age 13–31 y, median 19 y) were raised as females (further referred to as females/F) and 10 (age 0–24, median 9,5 y) as males (further referred to as males/M). Seven females had typical TS features with typical female external genitalia and short stature. Additionally, one child with ambiguous genitalia at birth was raised female. Four males were born with typical male external genitalia (EMS 12/12). Four showed milder (EMS 8–10/12) and 2 severe (EMS 5–6/12) degrees of undervirilisation. In male patients, features typically associated with TS, were frequently encountered. Detailed information on genotypes and phenotypes is represented in Table 1 and 2.

**Table 1:** Genotype and phenotype of patients with 45,X/46,XY (or variant) raised female.

Age£	Age Δ££	Karyotype	EMS	Associated features	Cardiovascular
21 y	15 y	45,X/46,XY	non-ambiguous F	short stature	bicuspid aortic valve
				short, webbed neck	prolonged QTc (465 ms)
				shield thorax	high heart rate (97/min)
				cubitus valgus	
				recurrent otitis	
				hypothyroidism	
30 y	9 y	45,X/46,XY	non-ambiguous F	short stature	prolonged QTc (471 ms)
				shield thorax	
				recurrent otitis	
38 y	12 y	45,X/46,XY	non-ambiguous F	short stature,**	normal
13 y	neonatal	45,X/46,X,i(Y)(q10)	non-ambiguous F	short stature	bicuspid aortic valve
				pectus excavatus	coarctation
				short metacarpals	
				dysmorphic auricles	
				recurrent otitis	
				impaired vision	
15 y	8 y	45,X/46,XY	non-ambiguous F	short stature	dilated left art. subclavia
				short, webbed neck	
				shield thorax	
				cubitus valgus	
				short metacarpals	
				short metatarsals	
				recurrent otitis	
				ADHD	
13 y	10 y	45,X/46,XY	non-ambiguous F	short stature	bicuspid aortic valve
				cubitus valgus	prolonged QTc (457 ms)
				recurrent otitis	high heart rate (92/min)
				neurocognitive disorder	dilated left art. subclavia
13 y	1y	45,X/46,X, mar(Yq12)	non-ambiguous F	short staturedysmorphic auriclesrecurrent otitishearing impairmenthorseshoe kidney	high heart rate (82/min)
26 y	neonatal	45,X/46,XY	virilisation*	short stature, **	right arteria lusoria

£ Age at the moment of examination in years,

££ Age at the moment of diagnosis.

* Clinical data are insufficient to calculate the EMS,

** more clinical data are not available.

EMS external masculinisation score.

**Table 2:** Genotype en phenotype of patients with 45,X/46,XY (or variant) raised male.

Age£	Age Δ££	Karyotype	EMS	Associated features	Cardiovascular
21 y	10 y	45,X/46,X,der(Y)	12	short stature,**	normal
24 y	7 y	45,X/46,X,idic(Y)(q11.21)	12	short stature	bicuspid aortic valve
				cubitus valgus	
				short metacarpals	
				short metatarsals	
				neurocognitive disorder	
11 y	10 y	45,X/46,X,idic(Y)(p11.32)	12	short stature	bicuspid aortic valve
				short neck	mildly dilated AoAsc
				shield thorax	prolonged QTc (465 ms)
				short metacarpals	high heart rate (95/min)
				short metatarsals	
				Scoliosis	
				ADHD	
				recurrent otitis	
1 y	prenatal	46,X,i(Y) (pter→q11:q11→pter)	12	cleft lip and palate	normal
10 y	1 y	45,X/46,XY	10	short stature	bicuspid aortic valve
				short, webbed neck	dilated AoAsc
				shield thorax	impaired systolic function
				short metacarpals	prolonged QTc (482 ms)
				short metatarsals	high heart rate (94/min)
				recurrent otitis	
19 y	1 y	45,X/46,XY	10	short stature,**	right arteria lusoria
3 y	prenatal	45,X/46,X,i(Y)(p10)	10	short stature	bicuspid aortic valve
				multicystic kidney	mildly dilated AoAsc
				recurrent otitis	
10 y	neonatal	45,X/46,XY	8	short stature	VSD
				short neck	bicuspid aortic valve
				shield thorax	dilated AoAsc
				short metacarpals	high heart rate (99/min)
				short metatarsals	
				horseshoe kidney	
5 y	neonatal	arrYq11.223q11.223/XO	6		normal
2 y	neonatal	45,X/46,X,isoYp	5	recurrent otitis	normal

£ age at the moment of examination in years,

££ age at the moment of diagnosis.

** more clinical data are not available.

AoAsc ascending aorta.

EMS external masculinisation score.

Fourteen patients (8 F, 6 M) received growth hormone for short stature. In four males, although short for age and (assigned) sex, growth hormone was not started yet in view of their young age.

### Clinical Examination and Cardiac History

Clinical cardiac examination was normal in all patients, except for the girl with previous cardiac surgery and a boy of 5 years old with a soft innocent murmur. None of them had any complaints or symptoms suggestive of underlying cardiovascular disease.

Five patients (4 F, 1 M) had previously visited a (paediatric) cardiologist. Routine echocardiography was performed in 3 females with TS and turned out to be normal in 2 patients, whereas one showed a bicuspid aortic valve. Additionally, one girl was previously diagnosed with a BAV and underwent neonatal cardiac surgery for coarctation of the aorta. One boy was known from infancy with a BAV and spontaneous closure of a VSD.

### Blood Pressure

Blood pressure was slightly elevated in 3 patients (2 F, 1 M), including the girl previously treated for coarctation.

### ECG

All except one (M, 1 year old) patient received a standard 12 leads ECG. In all, heart rate was within the normal range for age and (assigned) sex, although in 3 females and 3 males heart rate was at the upper limit of normal. A prolonged QTc was found in 5 patients (3 F, 2 M). QTc dispersion was longer than 60 ms in 10 patients (6 F, 4 M). No episodes of arrhythmia were reported. None of the patients received QTc prolonging medication.

### Echocardiography

Echocardiography was performed in all patients. A BAV was found in 8 (3 F, 5 M). None of the aortic valves was insufficient nor stenotic. Dimensions of AoRt and STJ were normal in all, but echocardiography failed to visualise the ascending aorta in 5 patients (4 F, 1 M). All received a cardiac MRI. Mild dilation (z-score 2.33–2.94) of the AoAsc was found in 3 patients (1 F, 2 M), severely dilation (Z-score 4.48–6.19) of the AoAsc in 2 (2 M). Inflow patterns over the mitral valve were normal in all patients. Systolic function was impaired (FS 26%) in a 10 year old boy, who also showed a severely dilated AoAsc.

### Cardiac MRI

MRI was performed in 12 patients. BAV was found in 4 (3 F, 1 M); in all, BAV was correctly diagnosed on echocardiography. Dimensions of AoRt and STJ were normal in all patients.

**Figure 1:**
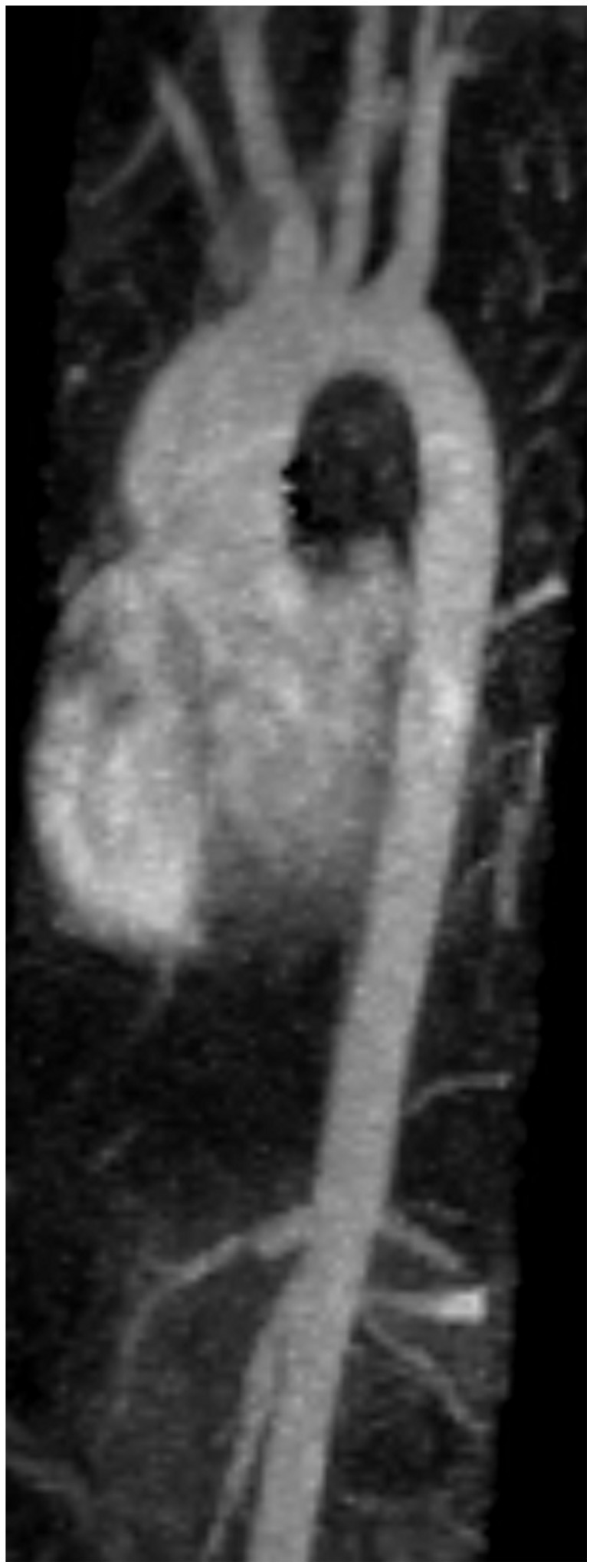
Maximum Intensity Projection image of contrast enhanced subtraction magnetic resonance angiography in arterial phase during intraveneous injection of Meglumine Gadoterate (Dotarem®). Pulmonary artery is cut out for clarity. Ascending aorta is dilated, normalizing over the arch; small calibre abdominal aorta. Normal branching of supra-aortic arteries.

Indexed AoAsc was smaller than 20 mm/m^2^ in 11 patients (8 F, 3 M) but was 25.9 mm/m^2^ in the boy who showed severe dilation on echocardiography (see Figure 1). The other boy diagnosed with severely dilated indexed AoAsc refused to have an MRI. All patients with dilated AoAsc had a BAV.

No patients were diagnosed with a persistent left superior caval vein or partial abnormal pulmonary venous return. MRI gave the additional diagnosis of a right sided arteria lusoria in two (1 F, 1 M) and a dilated left subclavian artery in two females.

Data on cardiovascular findings are summarized in Table 1 and 2.

## Discussion

Patients with 45,X/46,XY mosaicism and normal female external genitalia are often diagnosed with TS during childhood, due to short stature and eventually associated dysmorphic features. These girls enter a strict medical follow-up protocol, according to international guidelines, in order to prevent or treat an array of associated medical conditions such as short stature, conductive hearing loss, strabismus, celiac disease, hypothyroidism, metabolic disturbances and diabetes [12].

Although not always recognized, 45,X/46,XY boys often present with similar dysmorphic features, growth disturbances and comparable health problems later in life. As in TS, growth hormone therapy has proven to be effective in 45,X/46,XY boys with short stature [23]. In addition, 45,X/46,XY males frequently suffer from (mixed) gonadal dysgenesis and infertility and also renal pathology is reported [15]. In our series of 45,X/46,XY males, phenotypical features typically associated with TS were frequently encountered.

In TS, morbidity and mortality are high compared to the general population and cardiocirculatory disease accounts for the greatest excess mortality [4]–[6]. Cardiovascular disease is frequent and consists of structural heart defect, aortic dilation, hypertension and conductance disturbances. Congenital heart disease is common with a prevalence of 25 to 45%. Structural defects are mostly found at the left side of the heart with bicuspid aortic valve and coarctation being the most common [3], [7]–[9]. In the last 20 years, focus has been put on the dilation of the ascending aorta which can lead to dissection, disruption and sudden death at a relatively young age [4], [5], [21], [24]–[26]. Sporadic case reports describe coarctation and dilatation of the ascending aorta in males with 45,X/46,XY [16], suggesting similar underlying cardiovascular problems, but to our knowledge, this study is the first to investigate ECG, cardiac morphology and heart function in males with 45,X/46,XY as compared to 45,X/46,XY females.

The use of echocardiography in Turner patients is complicated by the disturbed body composition and short neck, and often yields inconclusive data [27]. In 5 of our 18 patients, visualisation was insufficient to obtain all information. Cardiac MRI was performed in all patients who did not require sedation to obtain full data acquisition (12/18, including all patients with suboptimal visualisation by echocardiography).

In our study population, bicuspid aortic valves were found in nearly 50% of the patients, with an almost equal distribution in males and females (3/8 F, 5/10 M), compared to 12.5% to 20% described in the general Turner literature [3], [7], [9], [28] and 1% in the general population [29]. None of the valves was stenotic nor insufficient.

Dilation of the aorta was only found at the site of the ascending aorta and was present in 4 boys (age 3 to 10 years old); all had a BAV. Two other boys were younger than 1 year old and had a normal aorta on echocardiography. However, longitudinal follow up is needed to rule out dilation in the future. In the 8 female patients, no AoAsc dilation was found.

MRI revealed the presence of a right arteria lusoria in 2/18 patients (1 F, 1 M). In the general population, the incidence of this anatomical variant is estimated at 0.4 to 2% [30]. Although described in the Turner population, no cases of abnormal venous return were found in our series.

In TS, ECG abnormalities are frequently seen both in girls and adults. Heart rates of TS patients tend to be higher compared to normal controls and QTc prolongation is more frequent (in 20 to 30%) [31]–[33]. Although for all patients in our series heart rate was within the normal limits, 6 patients (3 F, 3 M) showed a frequency at the upper limit. QTc interval exceeded 450 ms in 5 patients (3 F, 2 M) and QTc dispersion was abnormal in 10 (6 F, 4 M). In TS these finding are attributed to a disturbed sympathic- parasympathic balance [33]. The results of our study suggest that this imbalance is also present in 45,X/46,XY mosaic boys.

In our relatively young population, in office blood pressure measurement, as a primary screening tool, did only show mild hypertension in 3 (2F, 1M). Follow-up studies should include 24 hour ambulatory blood to rule out hypertension or loss of circadian rhythm.

The majority of both female and male patients included in our study were treated with growth hormone for short stature. Growth hormone however has not been associated with left ventricular hypertrophy, aortic dilation or hypertension in TS [34]–[36].

We found structural cardiovascular pathology in 6/10 males with 45,X/46,XY mosaicism as compared to 4/8 females. These problems were not only found in patients with ambiguous genitalia but to the same extent in males with apparently normal male genital development. However, those with cardiovascular pathology frequently showed multiple clinical features, typically associated with TS, as short neck, shield chest and short metacarpals. In the literature, a more sever phenotype in female Turner patients is associated with a higher prevalence of congenital heart disease and aortic dilation [9]. Unfortunately, detailed phenotypic data were not available for all participants. Large multicenter studies might be highly valuable to study the relation between cardiac abnormalities and Turner stigmata in 45,X/46,XY mosaicism.

The type of cardiovascular pathology encountered in TS does not always cause signs or symptoms. Therefore, recently, international guidelines advise a cardiac screening by an experienced cardiologist for every Turner patient at the time of diagnosis. For those with an apparent normal cardiovascular system and blood pressure, re-evaluation with MRI is advised at transition and subsequently every 5 to 7 years [12]. Besides 1 boy with an innocent murmur, none of our male patients showed clinical signs or symptoms that warranted a cardiac screening. We therefore recommend that the same guidelines on cardiac screening should be applied in all individuals, males and females alike, who are diagnosed with 45,X/46,XY mosaicism, irrespective of a normal clinical cardiac examination. Until more data are available on the natural progression of aortic dilation, repeated evaluation by echocardiography and MRI seems advisable.

No solid data currently exist to well-found guidelines on the prevention and treatment of dilation in Turner patients. The way of medical treatment, the optimal time to start and its benefits remain largely unknown. In most centres, current management is based on experience in Marfan patients who show similar aortic dilation. Medical treatment is started when the AoAsc diameter exceeds a z-score of +2. For Turner patients, Matura proposes to use an indexed AoAsc >20 mm/BSA as a cut-off value for abnormal dilation [21]. As a treatment, ACE inhibitors, betablockers and angiotensin-converting enzyme are prescribed but the usefulness of neither of them is proven. Also timing of surgical intervention is a subject of discussion. An indexed AoAsc ≥25 mm/BSA accounts for the 99th percentile in the Turner population, is associated with a very high risk for an acute aortic event and is currently used as an indication for surgical intervention [21]. High blood pressure poses an additional risk, both for dilation and atherosclerotic disease. Rigorous antihypertensive treatment is recommended by the classical therapies. In patients with prolonged QTc, medication with QTc prolonging property should be avoided. Because of the great similarity in cardiovascular pathology in 45,X/46,XY males and females, and due to the lack of prospective data, it currently seems logical to apply the same treatment strategy in these group as the one used in typical TS. In our cohort, we subsequently started treatment with an ACE-inhibitor in one and a beta-blocker in another boy.

Several limitations of the study should be taken into account. The number of patients included in the study is small. Patients were recruited via the Belgian Registry for Growth and Puberty problems and a university DSD outpatient clinic. This could cause a selection bias towards short boys and/or boys born with ambiguous genitalia. The study design does not allow to define the frequency of cardiovascular pathology in 45,X/46,XY males with a normal male phenotype, diagnosed in adult life. Follow-up studies should include a large sample of asymptomatic 45,X/46,XY males, and males diagnosed with mosaicism during work-up for infertility.

### Conclusion

Males with 45,X/46,XY mosaicism have the same type and frequency of cardiovascular pathology as 45,X/46,XY females with an incidence similar to what is found in classic TS patients. Dilation of the ascending aorta can occur early in life and might become life threatening. We advise cardiac screening and life-long monitoring in all males with 45,X/46,XY mosaicism according to the existing guidelines for TS.
